# Supplementary material for: Caffeoylquinic acid profiling: comparative analysis in yerba mate, Indian camphorweed, and stevia extracts with emphasis on the influence of brewing conditions and cold storage in yerba mate infusion
Source: PeerJ. 2024 May 6;12:e17250. doi: 10.7717/peerj.17250 (PMC11080990; doi:10.7717/peerj.17250)
Supplement: Supplemental Information 1 [file peerj-12-17250-s001.docx]

**Supplementary Table S1.** Information regarding the plant samples used in this study

| Plant name | Commercial brand | Package | Content | Production date | Expiration date | Commercial website (if any) |
| --- | --- | --- | --- | --- | --- | --- |
| *Ilex paraguariensis* (brand A) | Macher tea | Sachet | Dried leaves (100%) | 2019 | 2022 |  |
| *Ilex paraguariensis* (brand B) | Teblad | Sachet | Dried leaves (100%) | 2019 | 2022 | <https://www.teblad.com> |
| *Ilex paraguariensis* (brand C) | Cha Mate | Sachet | Dried leaves (100%) | 2019 | 2022 |  |
| *Stevia rebaudiana* (brand A) | Krittiya Garden | Sachet | Dried leaves (100%) | 2019 | 2022 | <https://www.krittiyagarden.com> |
| *Stevia rebaudiana* (brand B) | Baichaweingping | Sachet | Dried leaves (100%) | 2019 | 2022 |  |
| *Stevia rebaudiana* (brand C) | Kanthicha Nature | Sachet | Dried leaves (100%) | 2019 | 2022 |  |
| *Pluchea indica* (brand A) | Krittiya Garden | Sachet | Dried leaves (100%) | 2019 | 2022 | <https://www.krittiyagarden.com> |
| *Pluchea indica* (brand B) | Baichaweingping | Sachet | Dried leaves (100%) | 2019 | 2022 |  |
| *Pluchea indica* (brand C) | Kanthicha Nature | Sachet | Dried leaves (100%) | 2019 | 2022 |  |
