# Supplementary material for: Caffeoylquinic acid profiling: comparative analysis in yerba mate, Indian camphorweed, and stevia extracts with emphasis on the influence of brewing conditions and cold storage in yerba mate infusion
Source: PeerJ. 2024 May 6;12:e17250. doi: 10.7717/peerj.17250 (PMC11080990; doi:10.7717/peerj.17250)
Supplement: Supplemental Information 2 [file peerj-12-17250-s002.docx]

**Supplementary Table S2.** The limit of detection (LOD) and limit of quantification (LOQ) of caffeoylquinic acid standards used in this study

| Standard | Limit of detection (LOD) (μg/mL) | Limit of quantification (LOQ) (μg/mL) |
| --- | --- | --- |
| 1-CQA | 1.09 | 3.89 |
| 3-CQA | 6.34 | 19.23 |
| 4-CQA | 6.93 | 21.00 |
| 5-CQA | 10.53 | 31.93 |
| 1,3-diCQA | 9.26 | 28.07 |
| 1,4-diCQA | 8.06 | 26.77 |
| 1,5-diCQA | 7.77 | 23.56 |
| 3,4-diCQA | 5.45 | 16.51 |
| 3,5-diCQA | 16.79 | 50.89 |
| 4,5-diCQA | 6.11 | 18.52 |
