# Supplementary material for: Caffeoylquinic acid profiling: comparative analysis in yerba mate, Indian camphorweed, and stevia extracts with emphasis on the influence of brewing conditions and cold storage in yerba mate infusion
Source: PeerJ. 2024 May 6;12:e17250. doi: 10.7717/peerj.17250 (PMC11080990; doi:10.7717/peerj.17250)
Supplement: Supplemental Information 3 — Data are presented as the means ± standard deviations of three independent replicates. For each measured parameter, values in the same row followed by different superscript letters are significantly different (p < 0.05). [file peerj-12-17250-s003.docx]

**Supplementary Table S3.** Effect of storage under simulated home-refrigeration conditions (4 °C) on the color of yerba mate infusion.

| Sample | Fresh | Day 1 | Day 3 | Day 5 | Day 7 | Day 10 |
| --- | --- | --- | --- | --- | --- | --- |
|  | Lightness (*L*^*^) | | | | | |
| Infusion (normal) | 29.22 ± 0.56^a^ | 27.49 ± 0.87^a^ | 29.22 ± 0.79^a^ | 28.77 ± 0.33^a^ | 29.49 ± 0.46^a^ | 29.50 ± 1.01^a^ |
| Infusion (dark) |  | 28.01 ± 0.6^a^ | 27.38 ± 0.73^a^ | 28.39 ± 0.43^a^ | 29.46 ± 0.23^a^ | 28.44 ± 0.4^a^ |
| Redness/greenness (*a*^*^) | | | | | | |
| Infusion (normal) | 3.85 ± 0.19^a^ | 3.77 ± 0.8^a^ | 3.68 ± 0.28^a^ | 3.64 ± 0.52^a^ | 3.97 ± 0.09^a^ | 4.02 ± 0.66^a^ |
| Infusion (dark) |  | 3.93 ± 0.11^a^ | 3.92 ± 0.18^a^ | 3.66 ± 0.79^a^ | 3.98 ± 0.19^a^ | 3.66 ± 0.28^a^ |
| Yellowness/blueness (*b*^*^) | | | | | | |
| Infusion (normal) | 2.87 ± 0.11^a^ | 2.54 ± 0.11^a^ | 2.97 ± 0.23^a^ | 2.68 ± 0.11^a^ | 2.99 ± 0.23^a^ | 2.85 ± 0.32^a^ |
| Infusion (dark) |  | 2.99 ± 0.08^a^ | 3.01 ± 0.49^a^ | 3.07 ± 0.88^a^ | 2.88 ± 0.28^a^ | 3.11 ± 0.85^a^ |
| Color difference (Δ*E*) | | | | | | |
| Infusion (normal) |  | 0.36 ± 0.04^a^ | 0.35 ± 0.08^a^ | 0.34 ± 0.06^a^ | 0.35 ± 0.06^a^ | 0.32 ± 0.09^a^ |
| Infusion (dark) |  | 0.39 ± 0.05^a^ | 0.37 ± 0.07^a^ | 0.35 ± 0.07^a^ | 0.36 ± 0.04^a^ | 0.33 ± 0.08^a^ |

Data are presented as the means ± standard deviations of three independent replicates. For each measured parameter, values in the same row followed by different superscript letters are significantly different (*p* < 0.05).
