# Supplementary figures and images for: Caffeoylquinic acid profiling: comparative analysis in yerba mate, Indian camphorweed, and stevia extracts with emphasis on the influence of brewing conditions and cold storage in yerba mate infusion
Source: PeerJ. 2024 May 6;12:e17250. doi: 10.7717/peerj.17250 (PMC11080990; doi:10.7717/peerj.17250)

1-CQA

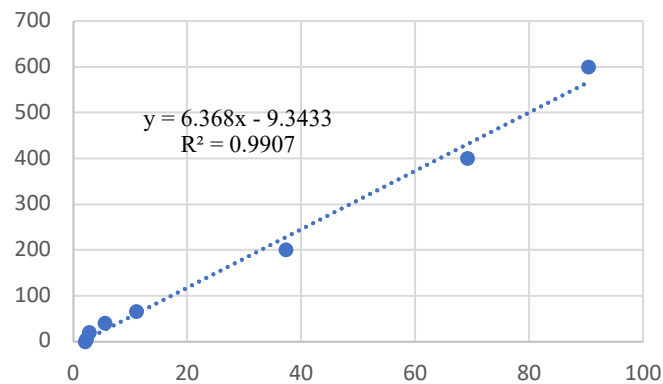

3-CQA

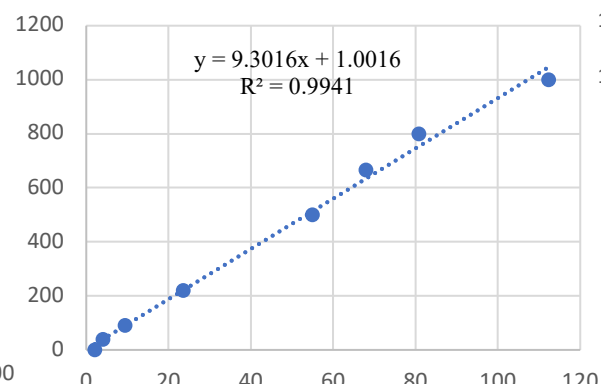

4-CQA

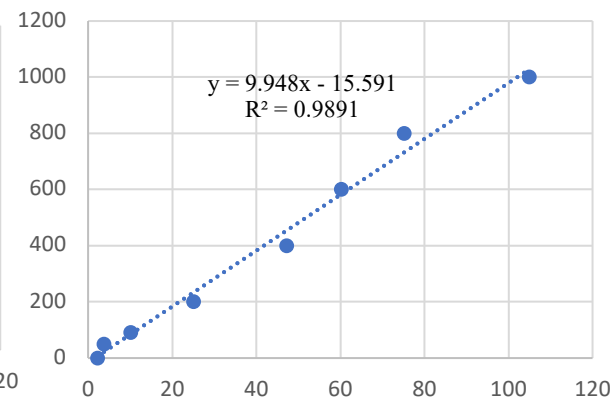

5-CQA

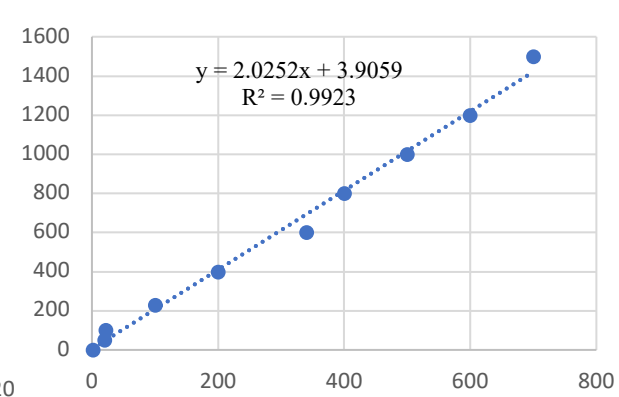

1,3-diCQA

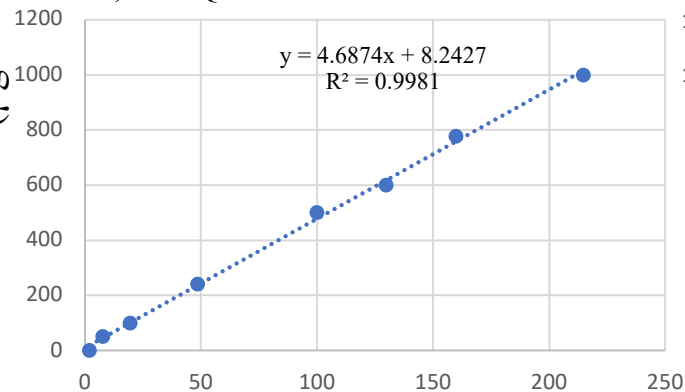

1,5-diCQA

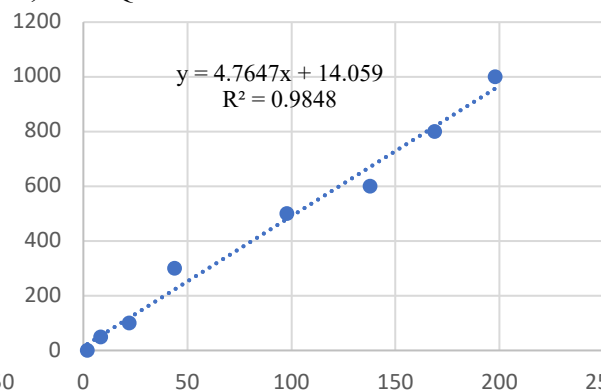

3,4-diCQA

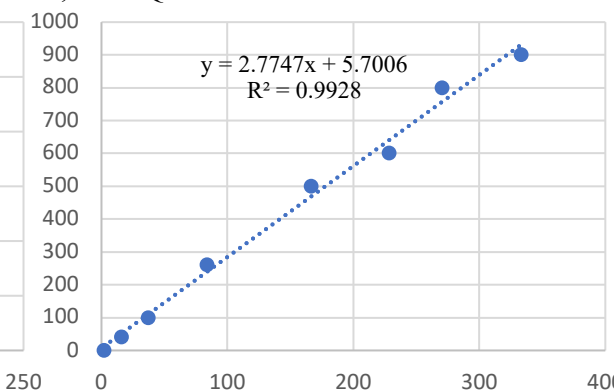

3,5-diCQA

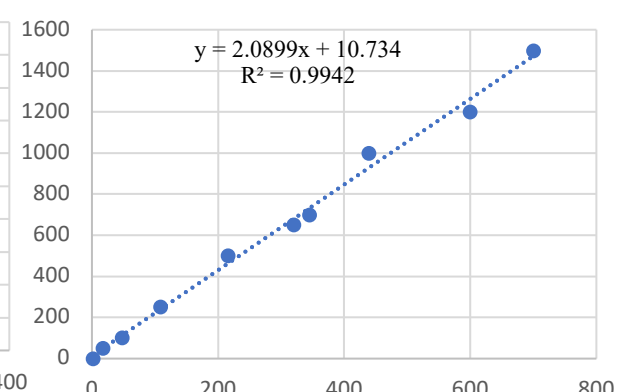

4,5-diCQA

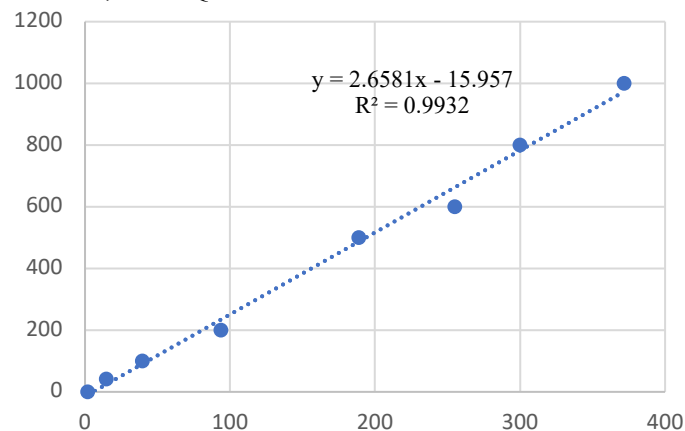

Area (mAU\*min)

Supplement: Supplemental Information 4 — Standard curves and equations of caffeoylquinic acid standards used in this study. [file peerj-12-17250-s004.pdf]

**A**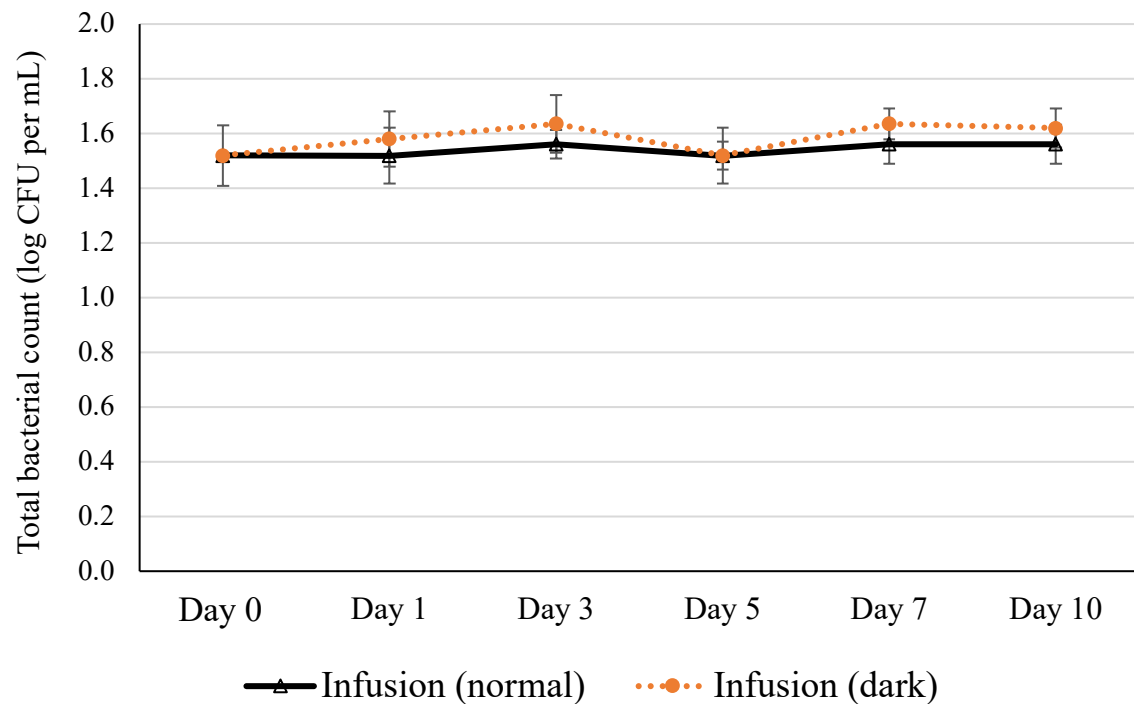**B**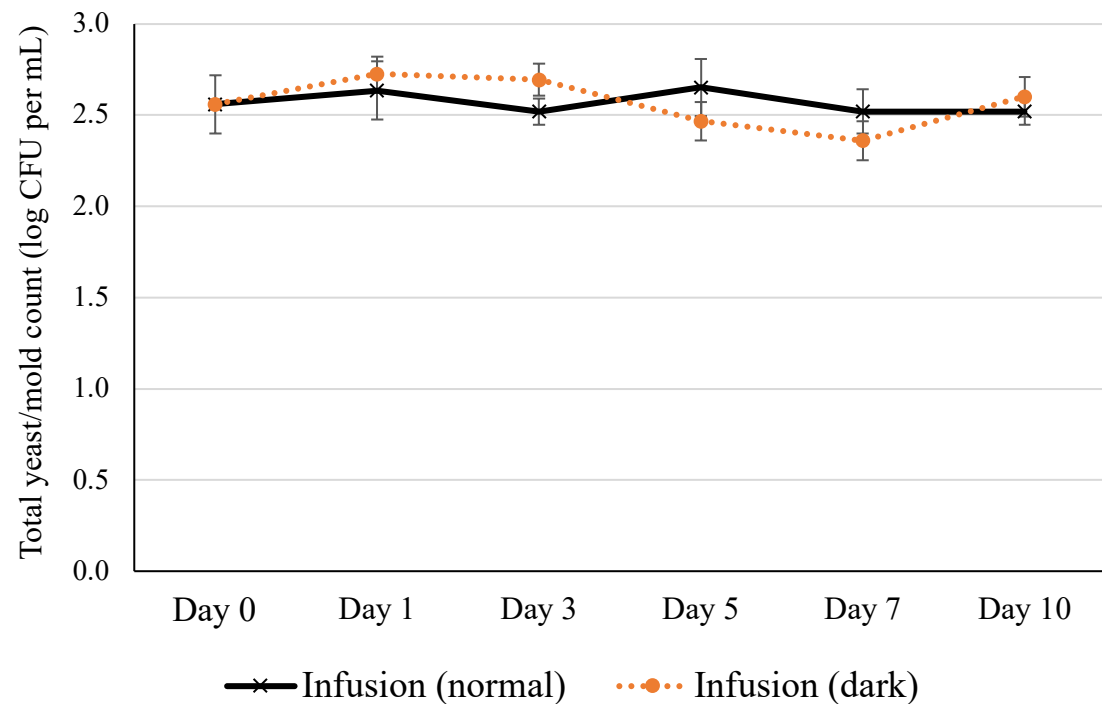

Supplement: Supplemental Information 5 — Effect of low-temperature storage on the total microbial count [(A) aerobic bacteria, (B) yeast and mold] of yerba mate infusions. Error bars represent means ± standard deviations (n = 3). No significant difference was observed compared to the sample at day 0 (control) (p < 0.05). [file peerj-12-17250-s005.pdf]
